# Manipulation of Elastic Instability of Viscoelastic Fluid in a Rhombus Cross Microchannel

**DOI:** 10.3390/polym14112152

**Published:** 2022-05-25

**Authors:** Meng Zhang, Zihuang Wang, Yanhua Zheng, Bifeng Zhu, Bingzhi Zhang, Xiaohui Fang, Wenli Shang, Wu Zhang

**Affiliations:** 1 School of Electronics and Communication Engineering, Guangzhou University, Guangzhou 510006, China; zhangmeng@gzhu.edu.cn; 2 School of Engineering and Applied Sciences, Harvard University, Cambridge, MA 02138, USA; 3 School of Physics and Material Science, Guangzhou University, Guangzhou 510006, China; 1919400068@e.gzhu.edu.cn (Z.W.); yhzheng@gzhu.edu.cn (Y.Z.); 2112119061@e.gzhu.edu.cn (B.Z.); zhang_bzh@gzhu.edu.cn (B.Z.); fangxiaohui@gzhu.edu.cn (X.F.)

**Keywords:** viscoelastic fluid, elastic instability, microfluidics, biological fluids

## Abstract

This paper reports the manipulation of elastic instability of the viscoelastic fluid in a rhombus cross microchannel (RCM) structure. The bistable instability and unsteady instability of the flow is firstly demonstrated in a standard cross microchannel (SCM) for reference. We then keep the bi-stable instability over a much wider injection rate range in the RCM, which is attributed to the stabilizing effect of the rhombus structure. A semi-bistable instability was also established in the RCM at a high enough injection rate. In addition, the unsteady elastic instability is realized in the RCM through an asymmetric injection rate condition.

## 1. Introduction

Microfluidic technology controls the fluid in micro-scale and has found wide applications in biochemical reaction [1,2], mass and heat transfer [3,4], polymerization [5], drug delivery [6], high-throughput sorting [7,8], health care [9], and other fields [10,11]. In microfluidics, the flow behavior is essential for developing different microfluidic chips such as microfluidic sensors and actuators [12]. For the common Newtonian fluid, the flow behavior is mainly characterized by the Reynolds number (*Re*), or the ratio of the inertial force to the viscous force in the fluid. Studies show that the flow instability raises in the Newtonian fluid only when *Re* of the flow is greater than 40 [13]. While in the microchannel, *Re* is usually less than 1 due to the micro-size restriction, leading to a negligible inertial effect, and the flow is in a stable creeping or laminar flow state and can be easily predicted.

However, most biological fluids, such as blood, saliva, cell fluid, and lymphatic fluid, are non-Newtonian viscoelastic fluids. These fluids usually contain elastic polymers which can cause elastic stress. Elastic stress is characterized by the Weissenberg number (*Wi*), defined as the ratio of the flow deformation rate to the polymer relaxation rate. When there is a velocity gradient in the flow, the polymer chain in the viscoelastic fluid will be stretched due to shear stress, and various uncommon flow behaviors can be induced such as the turbulent drag reduction at high *Re* [14,15], elastic turbulence at low *Re* [16,17], and secondary flow in lid-driven flow [18,19]. In macro-scale, the viscoelastic fluid exhibits special instability phenomena such as the pole climbing effect [20], tensile effect [21], and tubeless siphon effect [22]. In the microchannel with low *Re*, although inertia nonlinear effect is negligible, elasticity can trigger obvious nonlinear flow behavior [23] and even elastic turbulence [24]. Based on the above effects, viscoelastic fluid has been widely applied in different microfluidic circuit components for signal processing [25,26].

Due to the contribution of elastic instability, different microfluidic devices such as mixers [27,28], rectifiers [29,30], and memory and logic gates [26] have been well developed. Among the above microfluidic devices, cross-shaped microchannels have attracted intensive research attention due to their simple structure and ability to cause profound flow elastic instability [31,32]. For Newtonian fluids, the flow in the cross microchannel is perfectly symmetric when the injection at the two inlets is identical. Due to the symmetry of the flow, there is a stagnation point at the structure center and polymer in the fluid will be stretched surrounding the point for a sufficient period of time. The elastic stress generated by the extensional flow will result in elastic and nonlinear response of the fluid. Experiments and numerical calculation both verified the asymmetric instability of viscoelastic fluid in the cross microchannel [33]. In addition, quantitative studies of the flow pattern, pressure, and stress of viscoelastic fluid in the cross microchannel were carried out through different computational models [34]. It was proven that a bistable instability and an unsteady insatiability could be established successively as the *Wi* of the fluid increased [35]. The unsteady instability, which occurs in flows with a higher *Wi*, was later shown to be related to the “biregringent strant” effect resulting from the interaction of the fluid flowing from the zero velocity stagnation point to the outlet during long chain stretching of the polymer in the viscoelastic fluids [36]. Researchers also integrated the topology theory with the viscoelastic fluid flow model to optimize the size and shape of the cross microchannel, thereby reducing the minimum applied pressure value to obtain the instability [37]. The influence of the microchannel aspect ratio on the fluid instability in the cross microchannels was studied, which demonstrated a stabilizing effect by the side wall of the microchannel on the elastic instability [38]. In order to quantify the instability of viscoelastic fluid in cross microchannels, micro-PIV technology and birefringence imaging technology were applied to measure the flow velocity and study the change of anisotropy characteristics of polymer macromolecules in the fluid [39,40]. The above work reported robust realization of the elastic instability in the cross microchannel; however, the flexible manipulation of the elastic instability, which could be important for the instability control in different occasions, has not been studied to date.

**Figure 1:**
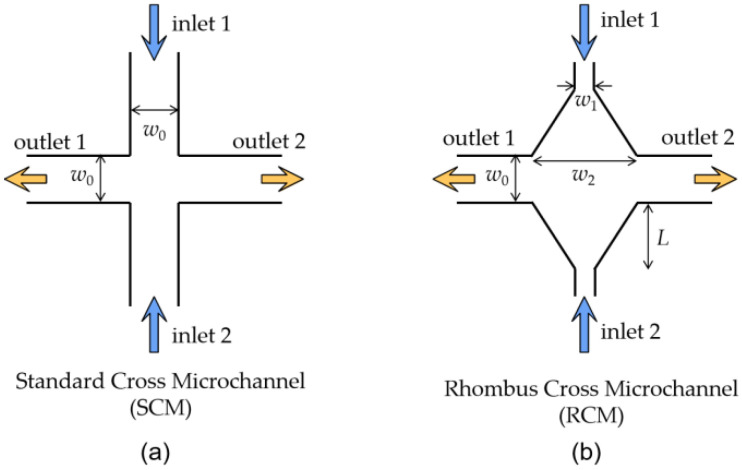
Schematic of (**a**) standard cross microchannel (SCM) and (**b**) rhombus cross microchannel (RCM).

In this paper, we will manipulate the elastic instability by controlling both the injected flow rate and the injection rate symmetry of the viscoelastic fluid in a rhombus cross structured microchannel. The rhombus cross structure is a cross structure with a tapered shape from the cross center to each of the four cross arms as illustrated in Figure 1. The flow pattern of the viscoelastic fluid in a standard cross microchannel (SCM) was first investigated for reference, which verifies the bistable instability and unsteady instability by simply increasing the injection rate of the fluid. The elastic instability was then manipulated by designing a rhombus cross microchannel (RCM). The polymers in the viscoelastic fluid will be pre-stretched in the extensional flow induced in the rhombus structure, which stabilizes the elastic inability behavior significantly. In addition, the unsteady elastic instability was manipulated through an asymmetric flow injection in the RCM.

## 2. Methods and Materials

The SCM and RCM structure for the elastic instability investigation are illustrated in Figure 1a,b. The inlets and outlets of the SCM has the same channel width *w*_0_ = 100 μm. For the RCM, it is also a symmetric cross microchannel structure. The two inlet channels have a narrow width *w*_1_ = 30 μm which expands linearly to *w*_2_ = 270 μm at the cross location with a tapering length *L* = 200 μm. The two outlets of the RCM have the same width *w*_0_ as that of the SCM. Both microchannels are designed at a height *h* = 100 μm. The channel length from inlets/outlets of each structure to the structure center are above 4 mm, which is enough for the full development of the flow.

The microchannels were fabricated using a standard soft lithography process. Firstly, a structure mold was made by the optical lithography process with a SU8-3000 photoresist (MicroChem, Round Rock, TX, USA). The photoresist layer in thickness of 100 µm is obtained on a Si wafer at a well-controlled spin rate recipe. The liquid polydimethylsiloxane (PDMS) at base and curing agent ratio of 10:1 was then poured on the Si wafer with the SU8 photoresist mold. An evacuating process was carried out to remove the bubbles in the PDMS liquid, followed by 4 h heating at 65 °C. The PDMS became a solid layer of 100 µm thickness and was peeled off from the Si wafer, which is then bonded with a flat PDMS layer to ascertain the microchannel structure.

**Figure 2:**
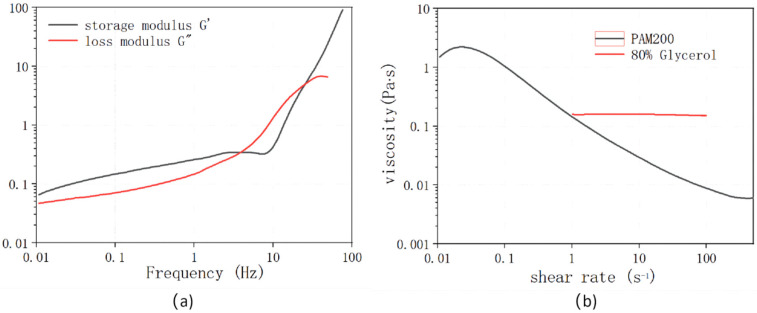
(**a**) Complex shear modulus of the PAM solution and (**b**) the viscosity of the PAM solution and the glycerol solution.

We used polyacrylamide (PAM) as the viscoelastic fluid for the elastic instability demonstration in the microchannel. The PAM used here has a molecular weight of 18 million (Polysciences, Warrington, PA, USA, polysciences18522-100) and a concentration of 200 ppm. The complex modulus of the PAM was measured using a rotational rheometer (Malvern, Discovery HR1) with cone-plate geometry. The cone has a diameter of 60 mm and angle of 2.006°. The complex shear modulus of the PAM was shown in Figure 2a. The storage modulus G’ is larger than the loss modulus G” at the lower frequency band, which indicates robust elastic property for the fluid. The viscosity of the fluid at different shear rates is shown in Figure 2b. The viscosity continuously decreases from ~10 Pa∙s to ~0.01 Pa∙s in the shear rate range of 0.1 s^−1^ to 100 s^−1^. For a reference, the flow of 80% glycerol solution is also measured, which has an almost constant viscosity at 0.1595 Pa∙s as shown in Figure 2b.

The flow pattern was visualized by feeding the fluid with fluorescent tracer particles (F8823, Life Technologies, Carlsbad, CA, USA, 2% solids) in one inlet and without tracer particles in the other inlet. The particles are fluorescent polystyrene micro-particles with a diameter of 1 µm and diluted in the fluids at a dilution ratio of 0.1 μL/mL. For description convenience, we name the inlet injected with tracer particle as the upper inlet, and the inlet without tracer particle as the lower inlet. The flow rates at the two inlets are controlled separately by two syringe pumps. The two outlets are open to the air. The flow pattern in the microchannel was recorded with an inverted epi-fluorescent microscope which focused on the center plane of the microchannel. The microscope was equipped with a high-speed camera operating at 20 fps, which captures the change of the flow pattern in real time.

## 3. Results and Discussion

**Figure 3:**
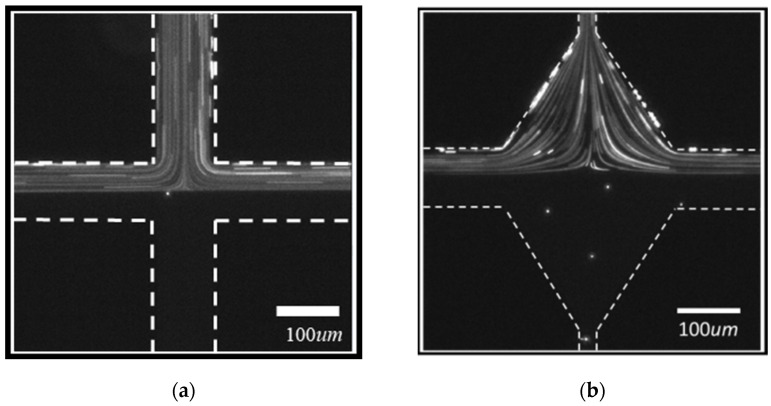
The glycerol flow pattern in the (**a**) SCM and (**b**) RCM at *Re* = 0.022.

For a reference for the elastic stress effect on the viscoelastic fluid in the microchannel, we first looked at the flow pattern of the Newtonian fluid in the SCM and the RCM. The glycerol solution with tracer particles was injected into the upper inlet, and the glycerol solution without the tracer particles was injected into the lower inlet. The injection rates at the two inlets were set to an equal value of *Q*. The flow can be characterized by its Reynolds number Re, which is defined as Re=ρUw/η, where ρ is the fluid density and equal to about 1 g/cm^3^. w equals *w*_0_ for SCM and equals (*w*_1_ + *w*_2_)/2 for RCM. U is the average flow velocity of the fluid and equals *Q*/(*hw*). η is the viscosity of the fluid and measured as about 0.16 Pa·s. For the same *Q*, the Reynolds numbers for the two microchannel structures are the same, which ranges from 0.0022 to 0.0448 when *Q* increases from 50 μL/hour to 1000 μL/hour in the experiments. During the change of the *Re*, the glycerol fluid in SCM and RCM both remain as steady flow patterns. Figure 3 shows the flow patterns at *Re* = 0.022 condition. As expected, the flow patterns in SCM and RCM are both symmetric and steady. The fluid with tracer particles flows out to both left and right side outlets in equal amounts. For each outlet, the fluid with tracer particles occupies half the cross section, while the fluid without tracer particles occupies the other half. By observing the streamline of the flow, we can also tell the fluid velocity at the cross center is 0, indicating a stagnation point at the cross center.

**Figure 4:**
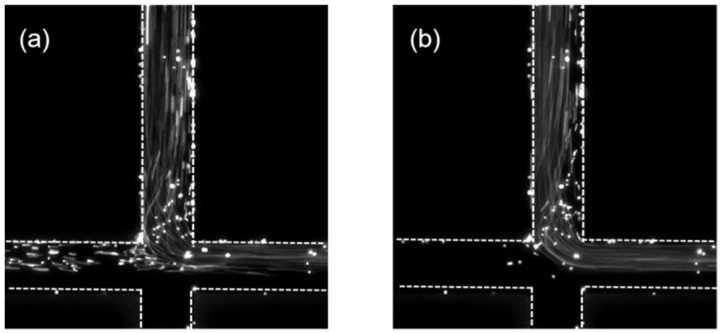
PAM flow pattern in the SCM at (**a**) *Wi* = 2.78 and (**b**) *Wi* = 5.56.

**Figure 5:**
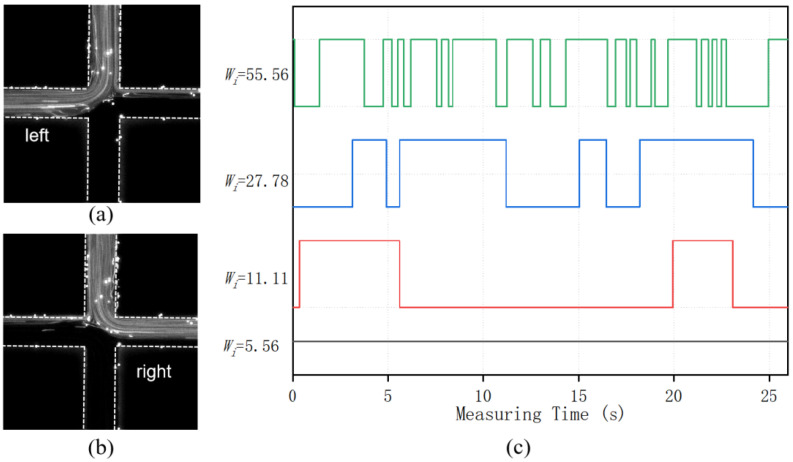
PAM flow pattern in the SCM at (**a**,**b**) two different instance at *Wi* = 11.11. (**c**) The flow bias direction in real time for PAM flow at different Wi numbers.

Then, we investigated the flow pattern of the viscoelastic fluid in the microchannels. In the PAM solution, polymers will be stretched and suppressed by the shear stress during the flow in the microchannel, exerting an elastic stress in the fluid, which will affect the flow behavior. The elasticity of the flow can be characterized by the Weissenberg number, which is defined as the viscous forces to the elastic forces. The number can be calculated as $Wi=\;\lambda \dot{\epsilon }$, where λ is the relaxation time of the fluid, referring to the characteristic stretch–relax time of the polymer, and was measured as ~0.1 s for the PAM solution of 200 ppm. $\dot{\epsilon }$ is the shear rate of the flow, equal to the ratio of the characteristic velocity to the characteristic length. For the SCM, $\dot{\epsilon }$ = *U*/(*w*_0_/2). Figure 4a shows the flow pattern of the PAM solution in the SMC at the injection rate of 50 μL/hour for both upper and lower inlets, with the corresponding *Wi* = 2.78. The flow pattern is symmetric and steady in the microchannel and therefore the fluid is in a stable state. This is similar with the flow pattern of the Newtonian fluid in the SCM, because the elastic stress in the PAM solution at this low *Wi* number is very weak and the flow is mainly dominated by the inertial effect of the fluid. As the injection rate at both inlets increased to 100 μL/hour, the corresponding *Wi* = 5.56 and the flow pattern of the PAM solution became asymmetric as shown in Figure 4b while remaining steady. The fluid with tracer particle was biased to the right outlet while the fluid without tracer particle was biased to the left outlet of the SCM. This bias direction is random depending on the initial state of the flow. However, once the bias is established, the flow pattern remained steady at this *Wi* number condition. Therefore, the flow is in a bi-stable state. When the injection rate at both inlets increased to 200 μL/hour with *Wi* = 11.11, the flow pattern of the PAM solution in the SCM started to change in real time. The fluid with tracer particles flows out through the left outlet for several seconds as shown in Figure 5a, and then swaps to the right outlet for the next several seconds as shown in Figure 5b. The swap continues during the measurement; thus, the flow is in an unsteady state. Figure 5c illustrates the real time change of flow direction of the PAM solution with tracer particles at different *Wi* number. When *Wi* = 5.56, the flow remained steady during the 25 s measuring time, which is presented by a straight black line. As the *Wi* increased to 11.11, the fluid with tracer particles started to flow out of the left outlets and right outlets alternatively, which forms an oscillation flow pattern in real time. As the injection rate at both inlets increased to 500 μL/hour and 1000 μL/hour, with corresponding *Wi* number at 27.78 and 55.56, the oscillation frequency of the flow pattern progressively increased as presented by the blue line and green line in Figure 5c. It is also can be seen that the oscillation occurs randomly.

The instability of the PAM solution in the SCM is due to the elastic stress in the flow. In the SCM, there is a stagnation point at the center of the cross where the flow velocity is equal to 0, and a strong velocity gradient is formed near the stagnation point. Due to the change of velocity gradient, significant elastic stress is generated on the polymer chain of the PAM solution. In the above measurements, both microchannel structure and injection rate are symmetric, while the flow pattern experiences different states at different *Wi* numbers. As *Wi* increases, the flow state transformed from the stable state to the bistable state and finally to the unsteady state. These phenomena stem from the elastic stress in the fluid, and are described as the elastic instability of the flow.

**Figure 6:**
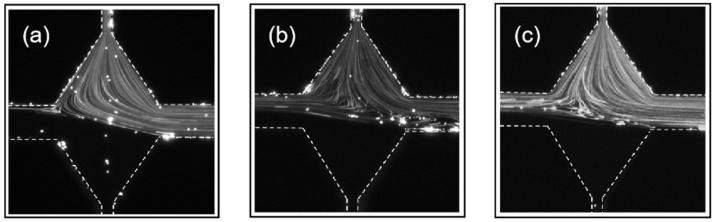
PAM flow pattern in the RCM at (**a**) *Q* = 50 μL/hour, (**b**) *Q* = 200 μL/hour, and (**c**) *Q* = 1000 μL/hour.

In the RCM, the polymer in the viscoelastic fluid will be stretched at the channel rhombus area first and then at the cross center of the microchannel. Therefore, the elastic stress of the PAM solution in the RCM will be different with that in the SCM, even at the same injection rate. In the expansion area, the shear rate of the flow is $\;\;{\dot{\epsilon }}_{1}$ = *U*_1_/(*L + w*_0_/2), where *U*_1_ is the flow rate at the inlet channel with width *w*_1_. The resulting Weissenberg number is *Wi*_1_. In the cross center, the shear rate is $\;{\dot{\epsilon }}_{2}$ = *U*_0_/(*w*_2_/2), where *U*_0_ is the flow rate at the outlet channel. The resulting Weissenberg number is *Wi*_2_. Figure 6 shows the flow patterns of the PAM solution in the RCM at different injection rates. When the injection rate at both inlets *Q* = 50 µL/hour, *Wi*_1_ is calculated as 1.85 and *Wi*_2_ as 1.03. As shown in Figure 6a, an asymmetry and steady flow pattern was observed so the flow is in the bistable state. Comparably, the flow of the PAM solution stays in the symmetric state at *Q* = 50 µL/hour and *Wi* = 2.78, and only reaches the bistable state at *Q* = 100 µL/hour and *Wi* = 5.56 in the SCM. Therefore, the RCM structure can realize the bistable state at a much lower injection rate and *Wi* number compared to the SCM. This should be due to the pre-stretch of the polymers in the expansion area, which induces extra elastic stress for the elastic instability. As the injection rate for the RCM gradually increases to 200 μL/hour with corresponding *Wi*_1_ = 7.41 and *Wi*_2_ = 4.12 (Figure 6b), and to 1000 μL/hour with corresponding *Wi*_1_ = 37.04 and *Wi*_2_ = 20.58 (Figure 6c), the flow pattern remains in the bi-stable state. Recall that the flow of the PAM solution in the SCM became unsteady when *Wi* is above 11.11. Therefore, we conclude that the RCM structure has a stabilizing effect on the elastic instability at high *Wi* condition, which should be due to the large rhombus area in the cross center. The above experiment indicates a wider *Wi* range for the bi-stable state in the RCM structure than that in in the SCM structure.

**Figure 7:**
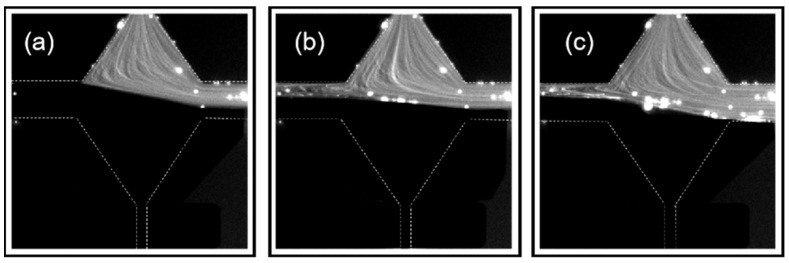
PAM flow pattern in the RCM at *Q* = 3000 μL/hour at three different instances. (**a**) fluid with tracer particles only flows to the right outlet; (**b**) fluid with/without tracer particles flows to both outlets; (**c**) fluid without tracer particles only flows to the left outlet.

As the injection rate increased to a very high value of 3000 μL/hour, the flow pattern of the PAM solution started to be time-dependent, which changes among the three states as shown in Figure 7a–c. At this injection rate, *Wi*_1_ = 111.11 and *Wi*_2_ = 61.73. In Figure 7a, the flow to the right outlet consists of both fluids, with and without tracer particles, while the flow to the left outlet only consists of the fluid without tracer particles. In Figure 7b, the flow in both the left and right outlets contains the fluid with and without tracer particles. In Figure 7c, the flow to the left outlet consists of both fluids with and without tracer particles, while the flow to the right outlet only consists of the fluid with tracer particles. The flow changes among the three states and the fluid with the tracer particle was kept biased to the right outlet during the experiment. The bias direction can also be swapped to the left outlet at the same injection condition depending on the initial bias state in repetitive experiments. We call this state a semi-bistable state, which stems from the balance between the high elastic stress in the fluids and the stabilizing effect of the rhombus structure.

**Figure 8:**
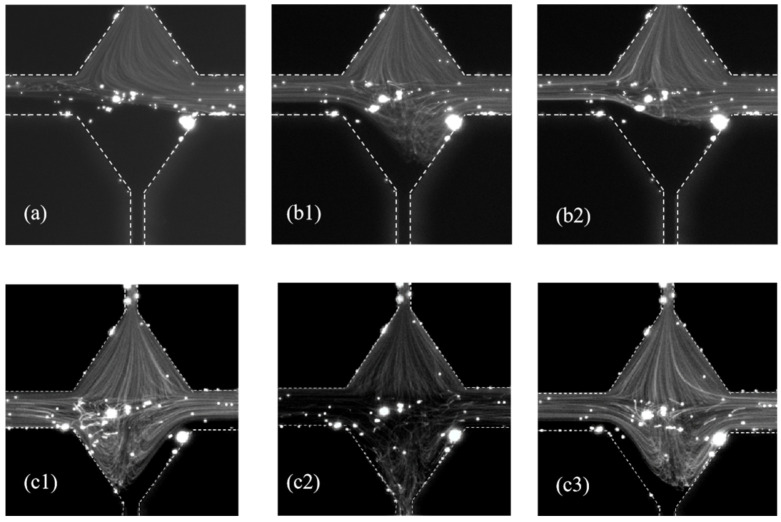
PAM flow pattern in the RCM with fixed *Q*_upper_ at 3000 μL/hour but different *Q*_lower_: (**a**) *Q*_lower_ = 2000 μL/hour, (**b1**,**b2**) *Q*_lower_ = 500 μL/hour, and (**c1**–**c3**) *Q*_lower_ = 200 μL/hour.

To further manipulate the elastic instability in the microchannel, we proposed an asymmetric injection rate at the two inlets by injecting the fluid with tracer particle at *Q*_upper_ = 3000 µL/hour in the upper inlets, while gradually decreasing the injection rate of the fluid without tracer particles *Q*_lower_ in the lower inlets. When *Q*_lower_ = 2000 μL/hour (Figure 8a), the flow pattern is left-right asymmetric while remain steady. Therefore, it is in the bi-stable state. When *Q*_lower_ decreased to 1000 μL/hour, the flow pattern changes between the state as shown in in Figure 8(b1,b2). The two states do not change the biased direction, but the boundary line between the fluids with and without tracer particles started to oscillate. This is similar to the semi-bistable state as described in Figure 7.

The flow patterns of the PAM solution at different instances are shown in Figure 8(c1–c3) when *Q*_lower_ decreased to 200 µL/hour and *Q*_upper_ remained at 3000 µL/hour. The boundary between the fluid with and without tracer particles oscillates forward to and backward from the lower part of the cross channel. The oscillation is due to different injection rate and thus elastic stress in the upper and lower part of the microchannel. The fluid without tracer particle flows out of the left channel at one moment, as shown in Figure 8(c1), and flows out of the right channel at another moment, as shown in Figure 8(c3). Therefore, the unsteady state is realized by introducing the asymmetric injection with two different injection rates, neither of which is high enough to induce the unsteady instability. The duration of the state for Figure 8(c1) or Figure 8(c3) can last for about 10 s~20 s. Figure 8(c2) shows the flow pattern of the transition state between that of Figure 8(c1,c3). It can be seen that the tracer particles can go into the lower part of the microchannel due to the unbalanced injection rate at the two inlets, which created chaos in the flow. This chaos only lasts for about 0.2 s and the flow quickly returns to the state in Figure 8(c1) or Figure 8(c3).

## 4. Conclusions

In a summary, we manipulated the flow instabilities of the viscoelastic fluid in a modified rhombus cross microchannel structure. It is demonstrated that the rhombus cross microchannel has a stabilizing effect on the viscoelastic flow which keeps the flow in the bistable state in a much wider flow rate range compared to that in a standard microchannel structure. A semi-bistable instability was also established at a high flow rate condition, in which the flow patterns change in real time while the flow bias direction remains the same. In addition, the unsteady elastic instability is demonstrated at an asymmetric injection condition with two different injection rates, neither of which is high enough to induce the unsteady instability in the rhombus cross microchannel.

## Data Availability

Raw data presented in this study are available on request from the corresponding author.
